# Comparative Genomic Analysis of *Mannheimia haemolytica* from Bovine Sources

**DOI:** 10.1371/journal.pone.0149520

**Published:** 2016-02-29

**Authors:** Cassidy L. Klima, Shaun R. Cook, Rahat Zaheer, Chad Laing, Vick P. Gannon, Yong Xu, Jay Rasmussen, Andrew Potter, Steve Hendrick, Trevor W. Alexander, Tim A. McAllister

**Affiliations:** 1 Agriculture and Agri-Food Canada Research Centre, Lethbridge, AB T1J 4B1, Canada; 2 Department of Large Animal Clinial Science, Western Colledge of Verterinary Medicine, University of Saskatoon, Saskatoon, Canada; 3 Laboratory for Foodborne Zoonoses, Public Health Agency of Canada, Lethbridge, Alberta, Canada; 4 Department of Biological Sciences, University of Lethbridge, Lethbridge, Alberta, Canada; 5 Vaccine and Infectious Disease Organization, Department of Veterinary Microbiology, University of Saskatchewan, Saskatoon, SK, Canada; Naval Research Laboratory, UNITED STATES

## Abstract

Bovine respiratory disease is a common health problem in beef production. The primary bacterial agent involved, *Mannheimia haemolytica*, is a target for antimicrobial therapy and at risk for associated antimicrobial resistance development. The role of *M*. *haemolytica* in pathogenesis is linked to serotype with serotypes 1 (S1) and 6 (S6) isolated from pneumonic lesions and serotype 2 (S2) found in the upper respiratory tract of healthy animals. Here, we sequenced the genomes of 11 strains of *M*. *haemolytica*, representing all three serotypes and performed comparative genomics analysis to identify genetic features that may contribute to pathogenesis. Possible virulence associated genes were identified within 14 distinct prophage, including a periplasmic chaperone, a lipoprotein, peptidoglycan glycosyltransferase and a stress response protein. Prophage content ranged from 2–8 per genome, but was higher in S1 and S6 strains. A type I-C CRISPR-Cas system was identified in each strain with spacer diversity and organization conserved among serotypes. The majority of spacers occur in S1 and S6 strains and originate from phage suggesting that serotypes 1 and 6 may be more resistant to phage predation. However, two spacers complementary to the host chromosome targeting a UDP-N-acetylglucosamine 2-epimerase and a glycosyl transferases group 1 gene are present in S1 and S6 strains only indicating these serotypes may employ CRISPR-Cas to regulate gene expression to avoid host immune responses or enhance adhesion during infection. Integrative conjugative elements are present in nine of the eleven genomes. Three of these harbor extensive multi-drug resistance cassettes encoding resistance against the majority of drugs used to combat infection in beef cattle, including macrolides and tetracyclines used in human medicine. The findings here identify key features that are likely contributing to serotype related pathogenesis and specific targets for vaccine design intended to reduce the dependency on antibiotics to treat respiratory infection in cattle.

## Introduction

*Mannheimia haemolytica* is an important bacterial pathogen of ruminants and the principal agent of bovine respiratory disease (BRD) in feedlot cattle, a condition responsible for an annual loss of over 3 billion US$ to beef producers worldwide [1]. The virulence and etiology of *M*. *haemolytica* is strongly associated with serotype. Of the 12 capsular serotypes identified, serotype 1 (S1) and serotype 6 (S6) are the most prevalent in bovine infection [2,3]. Serotype 2 (S2) is found frequently as a commensal in the upper respiratory tract of healthy cattle [4], but can produce pneumonia in ovines, frequently causing high mortality rates that have devastated wild and domestic sheep populations [5]. Little is understood about how genetic differences among serotypes contribute to pathogenesis in this species. Until recently, whole genome sequences were only available for one strain of S1 from a beef calf and two strains of S2, one of bovine and the other of ovine origin [6,7]. Although S1 is the principal serotype identified in North America, the prevalence of S6 in cattle suffering from BRD has been increasing [8].

Treatment of BRD in feedlot cattle relies heavily on antibiotics, and *M*. *haemolytica* is the primary target for many of these therapies. As a result, there is high risk for selection of antimicrobial resistant *M*. *haemolytica* within this setting. The recent isolation of pan-resistant *M*. *haemolytica* from beef calves [9,10] is of concern in terms of maintaining the therapeutic efficacy of antimicrobials for beef as well as the risk of transfer of antimicrobial resistance genes to zoonotic pathogens that can impact human health. Consequently, there is a need to develop less drug dependant strategies to control BRD. Veterinary vaccines can play a major role in the management of disease and in conferring protection against pathogens. However, the efficacy of currently available BRD vaccines is questionable [11].

Analysis of the genomic content of pathogenic bacteria can be used to identify novel protein targets in the aim towards developing more efficacious vaccines. The use of comparative genomic analyses can also highlight genetic features that contribute to pathogenicity, identify resistance mechanisms and help elucidate the evolution of pathogens at the genome level. Pan-genome analysis identifies the full complement of genes represented in a group of genome sequences and can provide important insights into the evolution of a species while identifying potentially important novel genes [12]. Extrapolation of pan-genome analyses can also highlight the global complexity of a bacterial species by allowing for prediction of the number of new genes that will be found with sequencing of additional strains [13]. Examination of genome architecture and prophage content can be used to identify genetic diversity and features like the CRISPR-Cas phage defence mechanism, self-transmissible integrative conjugative elements (ICEs) as well as genes that contribute to pathogenicity and survivability.

This study presents a comparative analysis of 11 *M*. *haemolytica* strains representing serotypes 1, 2 and 6 from cattle that were healthy, morbid or succumbed to BRD. Whole genome comparison of multiple serotypes allowed for the identification of multiple virulence factors that likely contribute to the pathogenesis of this species. The identification of ICEs that confer resistance to an array of antimicrobials illustrates the ability of this bacterium to circumvent current antimicrobial therapies. Multiple gene targets were identified that hold promise for further exploration as vaccine candidates.

## Results and Discussion

### Sequencing Statistics

**Fig 1:**
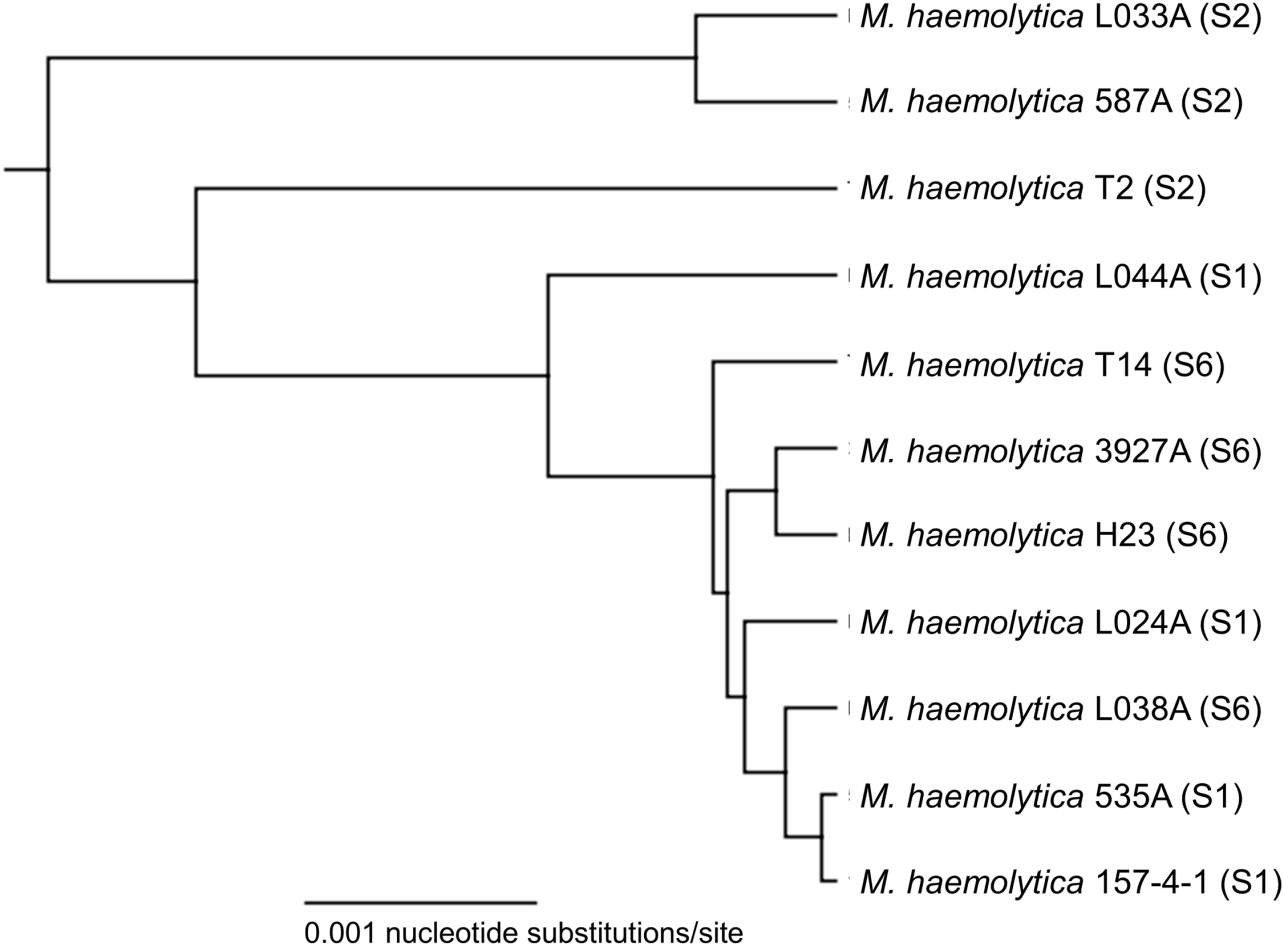
UPGMA phylogenetic tree based on whole genome sequence alignment of 11 *Mannheimia haemolytica* strains. The tree was generated from full genome alignment produced using Mauve in Geneious version 6.1.8 using Tamura-Nei distance correction with no specified outgroup. The scale length is equal to 0.001 nucleotide substitutions per site.

Sequencing of the 11 *M*. *haemolytica* strains resulted in high-quality draft genomes ranging from 2.43Mb to 2.60Mb, with assemblies containing 69–158 contigs (Table 1). Genomes of S1 and S6 strains are on average 100kb larger that S2 strains, a difference attributed to the presence of integrative conjugative elements and prophage. The percent nucleotide identity among the genomes ranges from 79.0% to 97.8% (Fig 1). Sequence diversity is highest amongst the three S2 genomes, ranging from 87.0% to 96.2% nucleotide identity.

**Table 1:** Features of 11 sequenced strains of *Mannheimia haemolytica*.

Strain	Accession No.	Serotype	Sample type	Animal status	Sampling location	Genomes size Mb	No. contigs	CDS	No. prophage (% of genome)	No. CRISPR spacers	ICE size kb (%GC)
*M*. *haemolytica* L024A	LFXX00000000	1	lung	Deceased	Texas	2.64	116	2768	8 (11.3)	14	70.6 (40.2)
*M*. *haemolytica* L044A	LFXY00000000	1	lung	Deceased	Nebraska	2.6	122	2714	5 (6.9)	15	81.1 (41.6)
*M*. *haemolytica* 157-4-1	LFYD00000000	1	nasopharynx	Healthy	Alberta	2.6	116	2715	7 (11.6)	14	48.6 (39.3)
*M*. *haemolytica* 535A	LFYB00000000	1	nasopharynx	Morbid	Alberta	2.58	158	2730	6 (10.5)	13	47.1 (39.3)
*M*. *haemolytica* T2	LFXW00000000	2	lung	Morbid	France	2.43	107	2448	2 (4.1)	8	n/a
*M*. *haemolytica* L033A	LFXZ00000000	2	lung	Deceased	Nebraska	2.57	128	2639	4 (8.2)	7	66.3 (40.2)
*M*. *haemolytica* 587A	LFYC00000000	2	nasopharynx	Healthy	Alberta	2.5	111	2547	5 (8.3)	4	n/a
*M*. *haemolytica* L038A	LFYA00000000	6	lung	Deceased	Alberta	2.6	134	2724	6 (10.9)	16	50.4 (39.1)
*M*. *haemolytica* T14	LFXV00000000	6	trachea	Morbid	France	2.56	101	2647	5 (9.2)	17	49.9 (39.1)
*M*. *haemolytica* H23	AOGP00000000	6	nasopharynx	Morbid	Alberta	2.6	69	2628	4 (7.6)	14	48.6 (39.3)
*M*. *haemolytica* 3927A	LFYE00000000	6	nasopharynx	Healthy	Alberta	2.52	105	2602	5 (7.4)	16	46.6 (39.4)

### Core, Dispensable and Pan-genome Analysis

Pan-genome analysis defines all coding sequences within a set of genomes and identifies core genes common to all strains, dispensable genes present in two or more strains, and strain specific genes that are unique to each isolate. Analysis of the different genome subsets can provide important insights into the evolution of a species and identify potentially important novel genes [12]. Pan-genome analysis can also determine the number of sequences in a genomic dataset that are sufficient to characterize the species as a whole [14]. The pan-genome can be defined as open, where the addition of new strains to the analysis results in the accumulation of additional unidentified genes, or as closed where sequencing of additional strains fails to identify additional strain specific genes. As a result, pan-genome analysis can be used to predict the number of whole genome sequences required to fully characterize a species [13,15].

**Fig 2:**
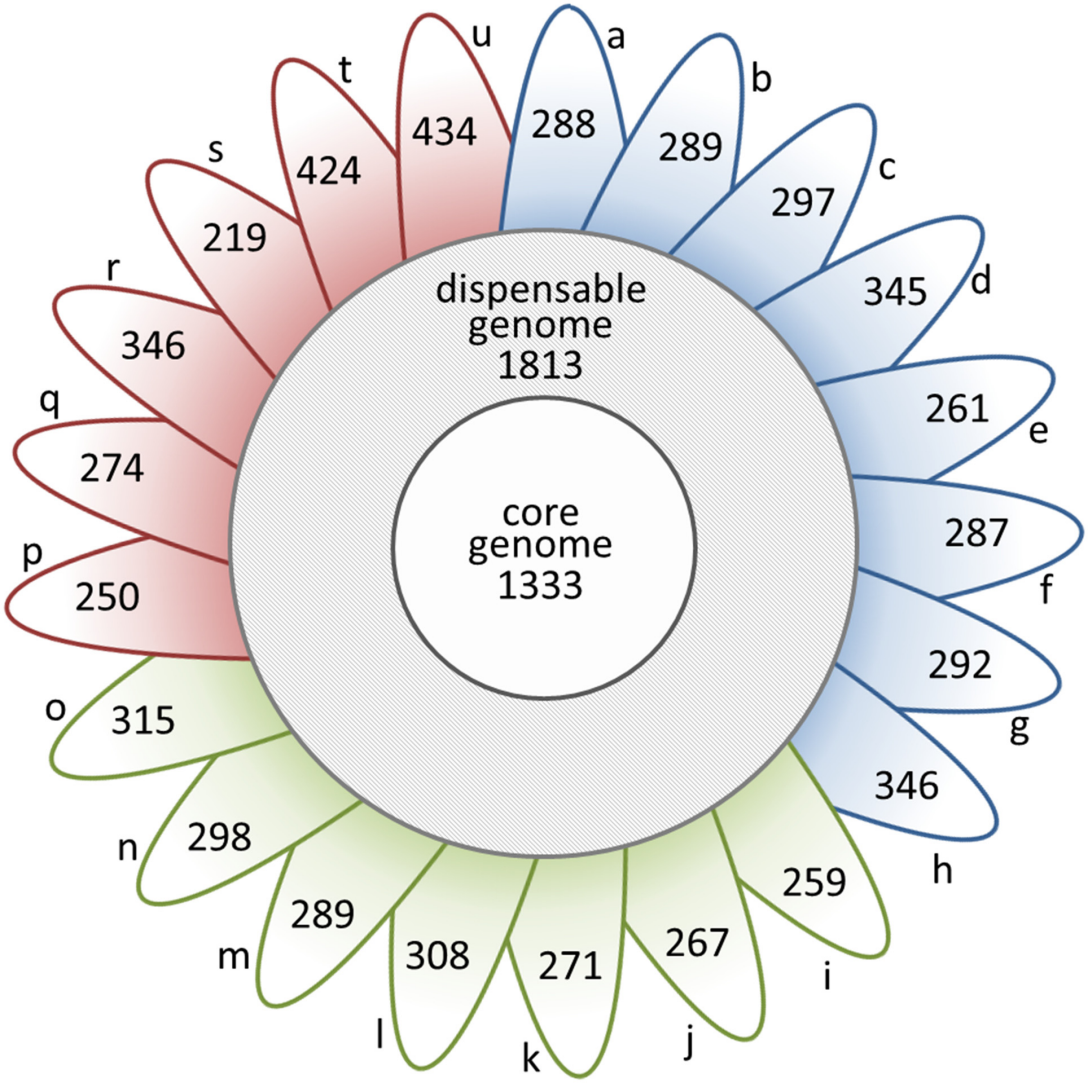
Size of the core genome and dispensable genome and number of strain unique CDS in 21 *Mannheimia haemolytica* genomes. Petals contain number of unique CDS per strain. Petals a-h represent the serotype 1 strains *M*. *haemolytica* L024A, *M*. *haemolytica* 157-4-1, *M*. *haemolytica* L044A, *M*. *haemolytica* 535A, *M*. *haemolytica* D153, *M*. *haemolytica* MhBrain2012, *M*. *haemolytica* D193, and *M*. *haemolytica* USDA-ARS-USMARC-183 respectively. Petals i-o represent the serotype 6 strains *M*. *haemolytica* T14, *M*. *haemolytica* H23, *M*. *haemolytica* 3927A, *M*. *haemolytica* L038A, *M*. *haemolytica* D174, D38, and *M*. *haemolytica* USDA-ARS-USMARC-185 respectively. Petals p-u represent the serotype 2 strains *M*. *haemolytica* 587A, *M*. *haemolytica* L033A, *M*. *haemolytica* T2, *M*. *haemolytica* D171, *M*. *haemolytica* D35 and *M*. *haemolytica* Bovine A2, respectively. Analysis based on 85% sequence identity across 90% length.

For the pan-genome analysis, the eleven isolates sequenced here were combined with an additional 10 present in public databases (S1 Table). Analysis of the resulting 21 *M*. *haemolytica* strains identified 9,507 orthologous groups, 1,333 belonging to the core genome (Fig 2), accounting for approximately 50% of coding sequences in each strain. As expected the core genome was comprised largely (76.5%) of coding sequences essential for cell function and survival (S1 Fig)[12]. The dispensable genome usually represents the bulk of the diversity in a species [14] and although here it contains genes associated with survival, maintenance, and defense, the majority (66.8%) are uncharacterized or hypothetical (S1 Fig). Genes that were found to be strain-specific are also largely (81.8%) uncharacterized, but many are associated with integrated prophage. Approximately 300 unique genes are present in each of the 11 strains (ranging from 219 to 434; Fig 2).

**Fig 3:**
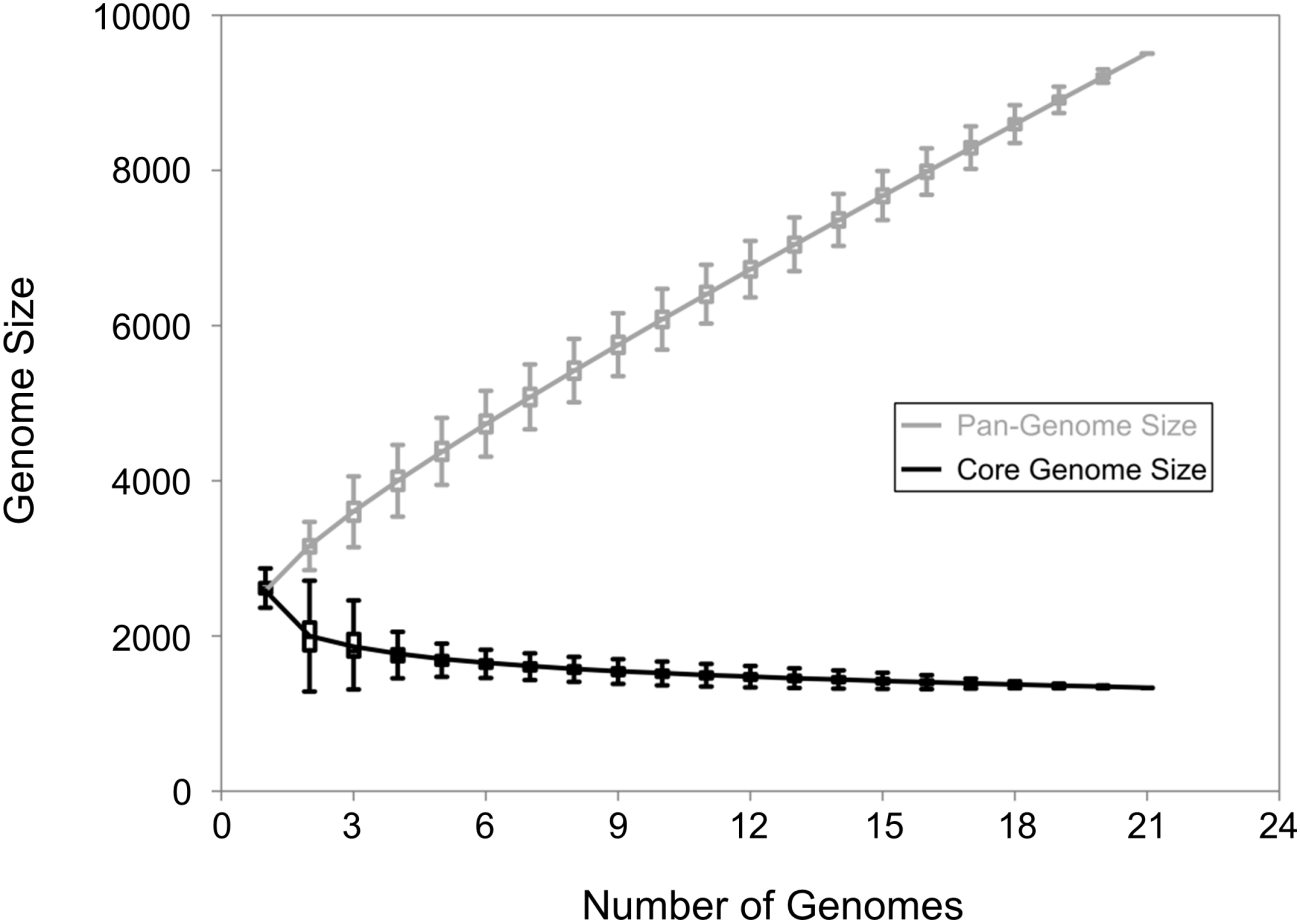
Pan-genome and core genome of 21 *M*. *haemolytica* isolates. For both the pan-genome and the core genome, the number of genes is plotted as a function of the number *n* of strains sequential added. The curve for the pan-genome represents the least-squares fit for the function y = Ax^B^ + C with the best fit obtained with a correlation r^2^ = 0.999 for A = 613.27 ± 1.08, B = 0.81, C = 2050.92 ± 10.41. The curve for the core genome represents the least-squares fit for the function y = Ae^Bx^ + C with the best fit obtained with a correlation r^2^ = 0.948 for A = 1444.89 ± 118.98, B = -0.32, C = 1409.54 ± 1.2.

**Fig 4:**
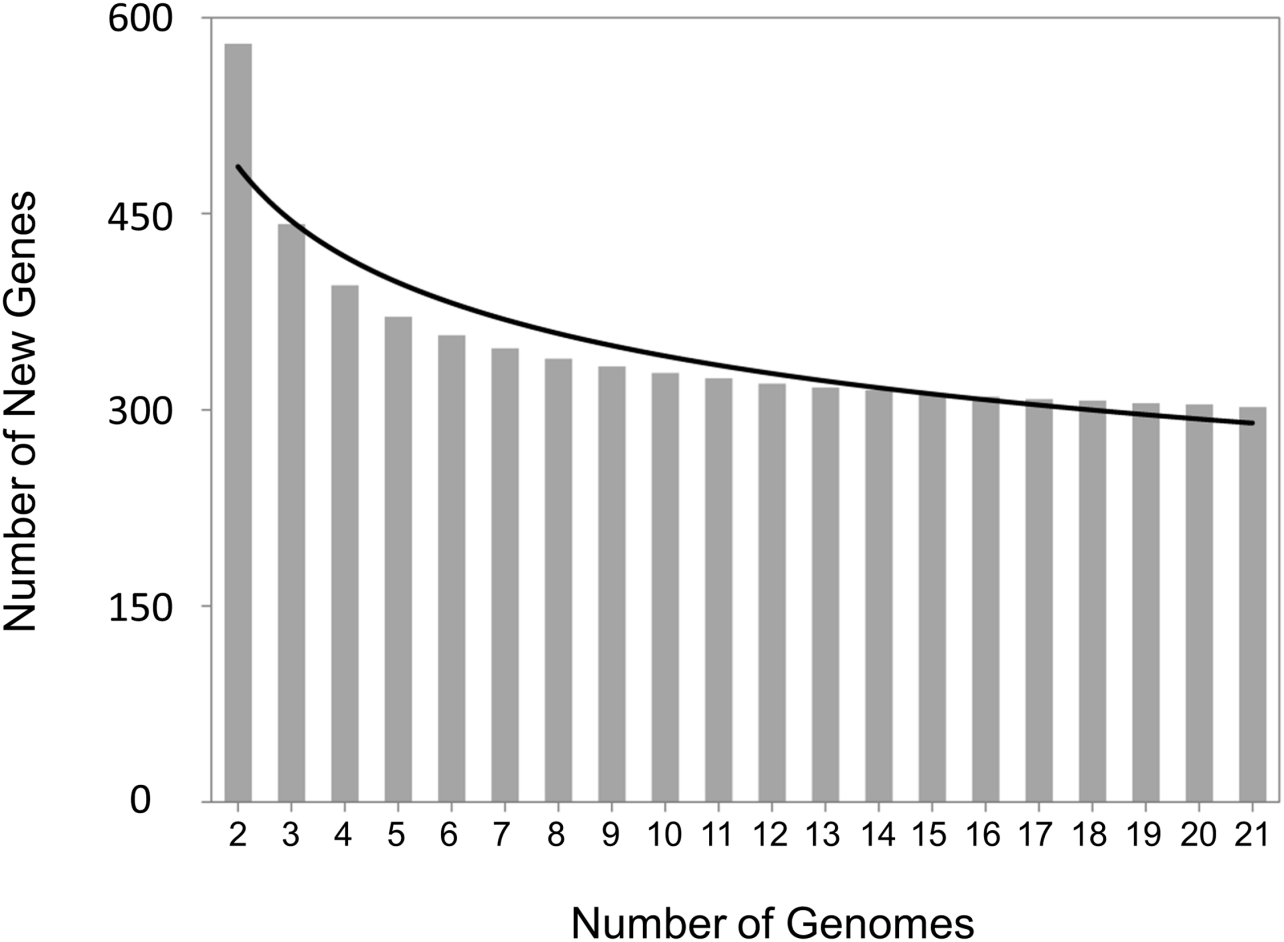
New gene discovery plot for pan-genome analysis of 21 *Mannheimia haemolytica* genomes. Bars represent the number of new genes as the function of the number n or strains sequentially added. The curve represents the least-squares fit for the function y = Ax^B^ with the best fit obtained with a correlation r^2^ = 0.875 for A = 566.17 ± 3.16, B = -0.22. The extrapolated number of new genes expected after the number of genomes increases to 100 is 123 new genes.

**Fig 5:**
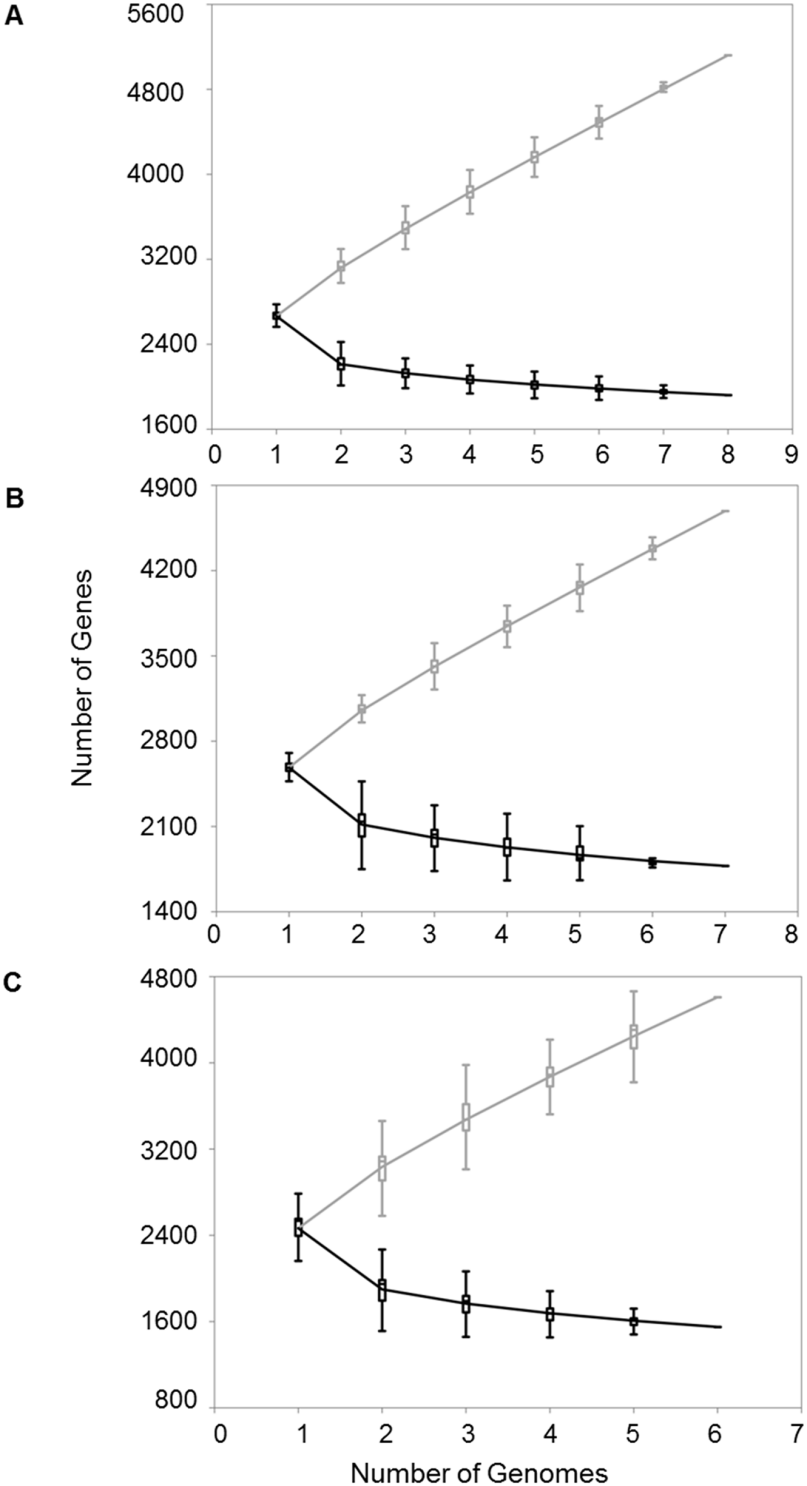
Pan-genome and core genome of 21 *M*. *haemolytica* isolates. For both the pan-genome (grey) and the core genome (black), the number of genes is plotted as a function of the number *n* of strains sequentially added. Panel A: serotype 1 strains, n = 8. Panel B: serotype 6 strains, n = 7. Panel C: serotype 2 strains, n = 6. The curve for the S1 pan-genome represents the least-squares fit for the function y = Ax^B^ + C with the best fit obtained with a correlation r^2^ = 0.999 for A = 572.81 ± 4.45, B = 0.79, C = 2104.32 ± 19.12. The curve for the S1 core genome represents the least-squares fit for the function y = Ae^Bx^ + C with the best fit obtained with a correlation r^2^ = 0.976 for A = 1549.09 ± 90.55, B = -0.81, C = 1964.34 ± 14.87. The curve for the S6 pan-genome represents the least-squares fit for the function y = Ax^B^ + C with the best fit obtained with a correlation r^2^ = 0.999 for A = 652.62 ± 8.5, B = 0.73, C = 1942.33 ± 25.16. The curve for the S6 core genome represents the least-squares fit for the function y = Ae^Bx^ + C with the best fit obtained with a correlation r^2^ = 0.983 for A = 1601.51 ± 92.41, B = -0.74, C = 1806.86 ± 19.97. The curve for the S2 pan-genome represents the least-squares fit for the function y = Ax^B^ + C with the best fit obtained with a correlation r^2^ = 0.999 for A = 944.93 ± 10.83, B = 0.65, C = 1527.44 ± 23.12. The curve for the S2 core genome represents the least-squares fit for the function y = Ae^Bx^ + C with the best fit obtained with a correlation r^2^ = 0.988 for A = 2123.13 ± 68.56, B = -0.88, C = 1579.86 ± 5.88.

The pan-genome of all 21 *M*. *haemolytica* strains is open, continuing to increase by approximately 286 genes with each strain added to the analysis (Figs 3 and 4). Extrapolation predicts that even after the addition of 100 genomes, 123 unique genes would be added with each new genome. Classification by serotype showed that both S1 (n = 8), S6 (n = 7) and S2 (n = 6) pan-genomes remained open (Fig 5A, 5B and 5C). The pan-genomes of S1 and S6 strains are expected to increase with each new strain by 305 and 295 genes, respectively. Unfortunately, the number of S2 strains available for analysis was too low to predict the number of new genes with each additional strain.

Open pan-genomes are frequently observed in bacterial species that inhabit diverse ecological niches, have complex lifestyles and are prone to horizontal gene transfer (HGT) [13,16]. In contrast, closed pan-genomes exist in bacterial species that occupy isolated niches or where recent divergence has led to limited genetic diversity within the gene pool [13]. The genome of *Bacillus anthracis* is an excellent example of a closed pan-genome as sequencing of four strains fully characterizes this species [14]. *Mannheimia haemolytica* inhabits a complex niche, can exhibit both commensal and pathogenic phenotypes, and contains many serotypes with variable amounts of mobile genetic elements (MGE) [5,7, 17]. Thus, it was anticipated that the pan-genome of this species would be open.

### Virulence Factors

*Mannheimia haemolytica* has multiple virulence factors that have been extensively studied and reviewed [18]. These include multiple fimbriae, adhesions, outer membrane proteins [19], neuraminidases [20], transferrin-binding proteins and a capsular polysaccharide [2]. One of the most important elements of *M*. *haemolytica* pathogenesis is a secreted leukotoxin of the RTX repeat family that exhibits cytolytic activity against bovine leukocytes [21]. We compared 72 genes with a known role in virulence, with the vast majority exhibiting >95% sequence identity among all 11 *M*. *haemolytica* genomes (S2 Table). Genes with sequence identities <95% included components of the transferrin-iron uptake system *tbpA* and *tbpB*, *lktA*, *lktC*, a surface antigen serotype-specific antigen 1 *ssa1*, the heme sequestering hemophore *huxA*, the S-ribosylhomocysteinase *luxS*, the outer membrane protein *plpE*, and *wecC* associated with lipopolysaccharide (LPS) synthesis. Sequence variations between serotypes for *tbpA* and *tbpB* [22], *lktA* and *lktC* [17,23,24] and *plpE* [25] have been documented previously. In all of these cases, sequence diversity was observed largely within the S2 strains, with genes in S1 and S6 strains being nearly identical.

### Mobile Genetic Elements

Horizontal gene transfer (HGT) through transduction, transformation and conjugation is the primary means for prokaryotic organisms to obtain new genetic material [26]. Mobile genetic elements (MGE) including plasmids, phages, genomic islands or genomic modules can transfer DNA that may alter the pathogenicity of an organism through the provision of toxins and antimicrobial resistance genes. They may also provide factors that regulate virulence gene expression, influence adhesion or enhance immune evasion [27]. As a result HGT plays an important role in the evolution, maintenance and transmission of virulence genes and the development and spread of antimicrobial resistance [28]. Two types of MGE, prophage and integrative conjugative elements (ICEs) were identified in *M*. *haemolytica* genomes.

### Prophage

**Fig 6:**
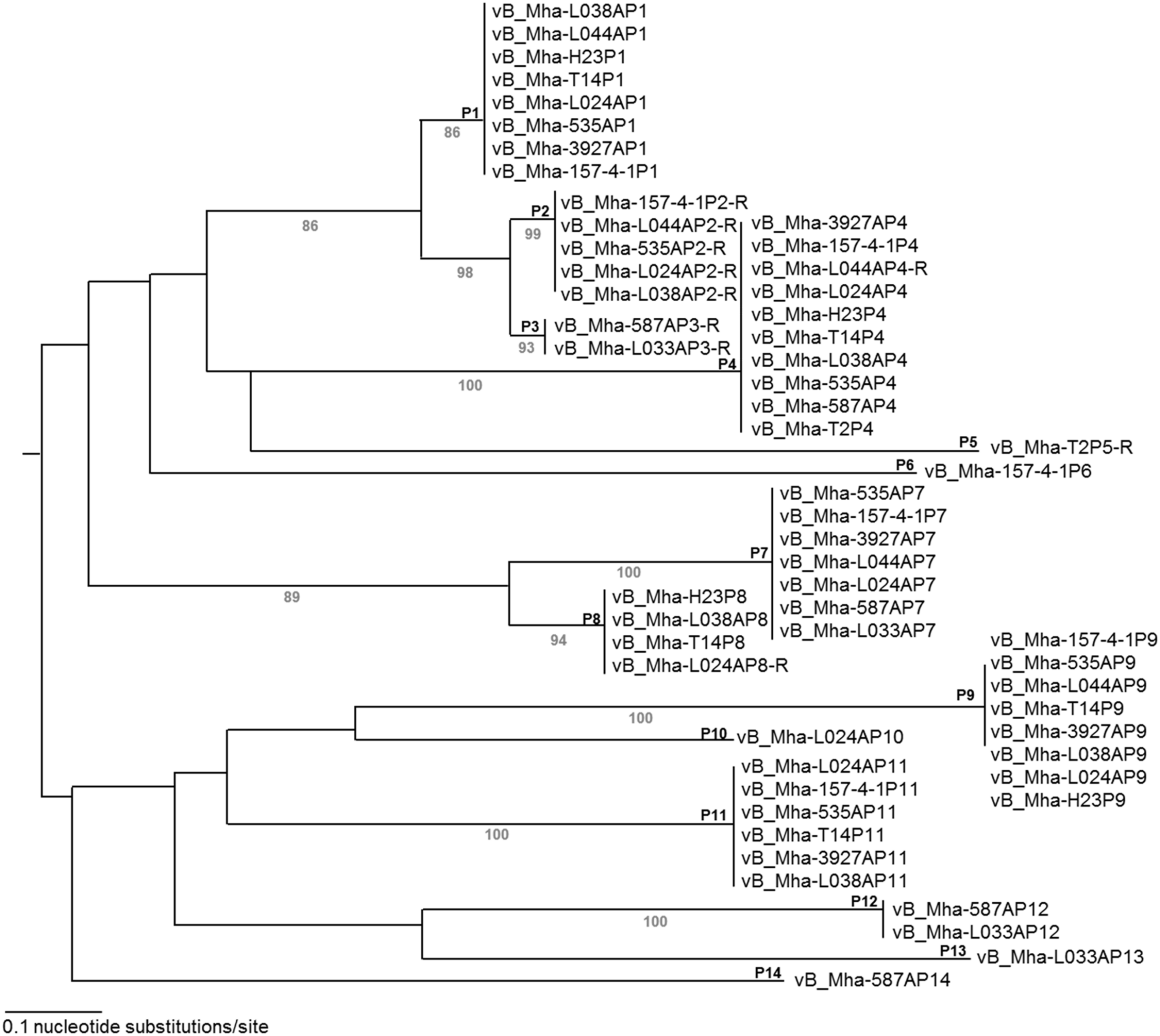
Phylogenetic analysis of prophage found in *M*. *haemolytica*. The tree was constructed by neighbour-joining method with the MAFFT program. Bootstrap values (>50) representing 100 sampling replicates are in grey located below branches with the phage cluster numbers adjacent to corresponding nodes above the branches. The scale length is equal to 0.1 nucleotide substitutions per site.

Prophage contribute to genetic diversity in bacterial genomes and act as vectors for virulence factors including extracellular toxins, adhesions, modulators of host activity, mitogenic factors and proteins that alter antigenicity [29,30]. Prophages account for 4.1–11% of the *M*. *haemolytica* genomes, with a total of 57 intact prophages present across the 11 strains. These cluster into 14 profiles based on nucleotide similarity (Fig 6; Table 2). Overall, S2 genomes contain fewer intact prophages (2–5) than S1 (5–8) or S6 (4–6) genomes. Clusters P1, P2, P3, P4, P7, P8, P13 and P14 resemble phage from the Siphoviridae family whereas P6, P9, P10, P11, and P12 clusters resemble the Myoviridae family. Prophages vB_Mha-L024AP10 and vB_Mha-157-4-1P6 cluster independently, and while these elements contained phage components they are comprised of multiple contigs and their lack of alignment to the reference strain suggest they may not be true prophages.

**Table 2:** Metadata for prophage identified within the genomes of 11 strains of *Mannheimia haemolytica*.

						Host strain of *Mannheimia haemolytica* and prophage length in kb (No. CDS):										
Cluster	Max length (kb)	Percent similarity	Spacer^a^	Toxin-antitoxin	Prophage family	587A	L033A	T2	L038A	H23	T14	3927A	154-7-1	L044A	L024A	535A
P1	42.7	95.3	-	RelB/E	Siphoviridae	-	-	-	38.5 (36)	41 (39)	38.9 (38)	39 (38)	30.9 (36)	31.5 (36)	39.9 (39)	34.7 (35)
P2	56.1	92.2	31	-	Siphoviridae	-	-	-	51.1 (64)	-	-	-	42.9 (62)	42.1 (61)	38.7 (59)	53.9 (64)
P3	43.3	84	8, 28, 29, 30, 31	HigA/B	Siphoviridae	52.1 (81)	104.1 (148)	-	-	-	-	-	-	-	-	-
P4	97.9	60.7	11, 12, 15	-	Siphoviridae	38.8 (45)		44.6 (52)	76.1 (82)	77.5 (83)	76.9 (82)	27.6 (29)	76.1 (81)	65.2 (82)	76.2 (82)	75.1 (84)
P5	54	-	-	HigA/B	Siphoviridae	-	-	53.9 (62)	-	-	-	-	-	-	-	-
P6	20.5	-	-	HipA/ B	Myoviridae	-	-	-	-	-	-	-	20.4 (26)	-	-	-
P7	54	67.1	3, 6, 17, 25	HicB/C	Siphoviridae	30.3 (51)	35.4 (48)	-	-	-	-	37.2 (52)	48.8 (52)	25.5 (43)	27.6 (48)	31 (46)
P8	39.1	59.7	3, 6, 16, 25	HigA	Siphoviridae	-	-	-	32.5 (51)	37.4 (51)	34.4 (46)		-	-	22.3 (24)	-
P9	47.1	94.5	-	-	Myoviridae	-	-	-	46.3 (60)	41.8 (51)	41.9 (57)	42 (57)	46 (60)	46.2 (61)	45.2 (61)	40.9 (56)
P10	14.1	-	-	HipB	Myoviridae	-	-	-	-	-	-	-	-	-	14 (21)	-
P11	47.5	85.1	26, 27	HipB	Myoviridae	-	-	-	38.3 (51)	-	43.4 (57)	40.1 (54)	34.8 (48)	-	35 (49)	35 (49)
P12	43.3	84	-	-	Myoviridae	41.9 (58)	41.1 (56)	-	-	-	-	-	-	-	-	-
P13	29.3	-	-	HicB/C	Siphoviridae	-	29.3 (39)	-	-	-	-	-	-	-	-	-
P14	44.7	-	-	HigA	Siphoviridae	44.7 (47)	-	-	-	-	-	-	-	-	-	-

^a^CRISPR spacer found with >90% sequence identity to phage sequence

Complete (P1, P3, P5, P6, P7, P13) or partial (P8, P10, P11, P14) toxin-antitoxin systems occur in 10 of the 14 phage clusters identified (data not shown). Toxin anti-toxin systems are small genetic modules that consist of an operon coding for a stable toxin that inhibits cell growth and an unstable antitoxin that protects against the toxin’s effects [31,32]. Toxin-antitoxin systems are frequently carried on MGE such as phages and plasmids, contributing to maintenance of the element once it has integrated into the host [33]. When co-expressed, these components form a stable complex inhibiting the toxin’s activity. Free antitoxin is highly unstable and degrades quickly requiring its continued synthesis to inhibit the toxin’s function [33].

During phage infection, protein expression in the host can be slowed or arrested. If the infected host bacterium codes for a toxin-antitoxin system the liable antitoxin it produces is degraded, leaving the toxin to kill the cell, ultimately preventing the phage from replicating and spreading. In bacteria this phage defence mechanism is known as abortive infection. The toxin-antitoxin systems identified here are mainly associated with Siphoviridae-like prophages, although two Myoviridae-like prophages (P10 and P11) contain HipB antitoxin without its corresponding HipA toxin (Table 2). It is possible these are remnants of previously functioning systems or that the antitoxin component is being used by the prophage as a means to avoid abortive infection in the host given that both strains of *M*. *haemolytica* that contain P10 and P11 prophage code for the HipA toxin in other regions of their chromosome.

Multiple virulence associated genes were identified in the prophages, including a periplasmic chaperone *lolA* (P5), involved in outer membrane localization of lipoproteins, and *plp4* (P4), a lipoprotein with homology to OmpA. A peptidoglycan glycosyltransferase, *mtgA* (P5) with homology to penicillin binding protein is also present. A mutation within *mtgA* (P5) in *Brucella abortus* has previously been shown to attenuate virulence in this species [34]. The *sanA* (P2) gene was also detected, a gene coding for a protein that functions in murein synthesis but that has also has been implicated in vancomycin resistance [35]. The gene for a cytoplasmic protein UspA (P4, P14) was also identified which has been predicted to enhance cell survival during exposure to environmental stress [36].

### CRISPR-Cas

Nearly half of all bacterial species encode an adaptive immune system known as CRISPR/Cas (clustered regularly interspaced short palindromic repeats/CRSIPR associated proteins) [37,38], that protects against invading genetic elements like phages and plasmids [38]. Three major types and ten subtypes of CRISPR-Cas systems have been identified [38] all containing a CRISPR array localized near 4 to 20 CRISPR associated (Cas) genes. The CRISPR array consists of a leader sequence followed by numerous conserved direct repeat (DR) sequences, interspersed with variable spacer sequences [37]. Spacers are typically derived from viral or phage DNA [37,39] and are used by a suite of Cas proteins to prevent reinfection by these elements through a process called interference [38]. Cas proteins support the integration of spacers into the CRISPR array, the processing of the transcribed CRISPR array into mature crRNA that contain a spacer and a partial DR, and the targeting and cleavage of reinvading nucleic acids complementary to the spacer sequences [38]. The selection of protospacers (spacer precursors) from the invading nucleic acid is dependent on the presence of a protospacer adjacent motif (PAM), a short nucleic acid sequence located either downstream or upstream of the protospacer. Protospacer adjacent motifs are not incorporated into the spacer itself, enabling the CRISPR-Cas system to distinguish between target protospacer and the host CRISPR array [37]. In addition to its role in defence, CRISPR-Cas has also been implicated in gene expression and regulation of cellular processes including biofilm formation, lysogenization, spore formation, replicon maintenance and segregation, and DNA repair-recombination [37].

**Fig 7:**
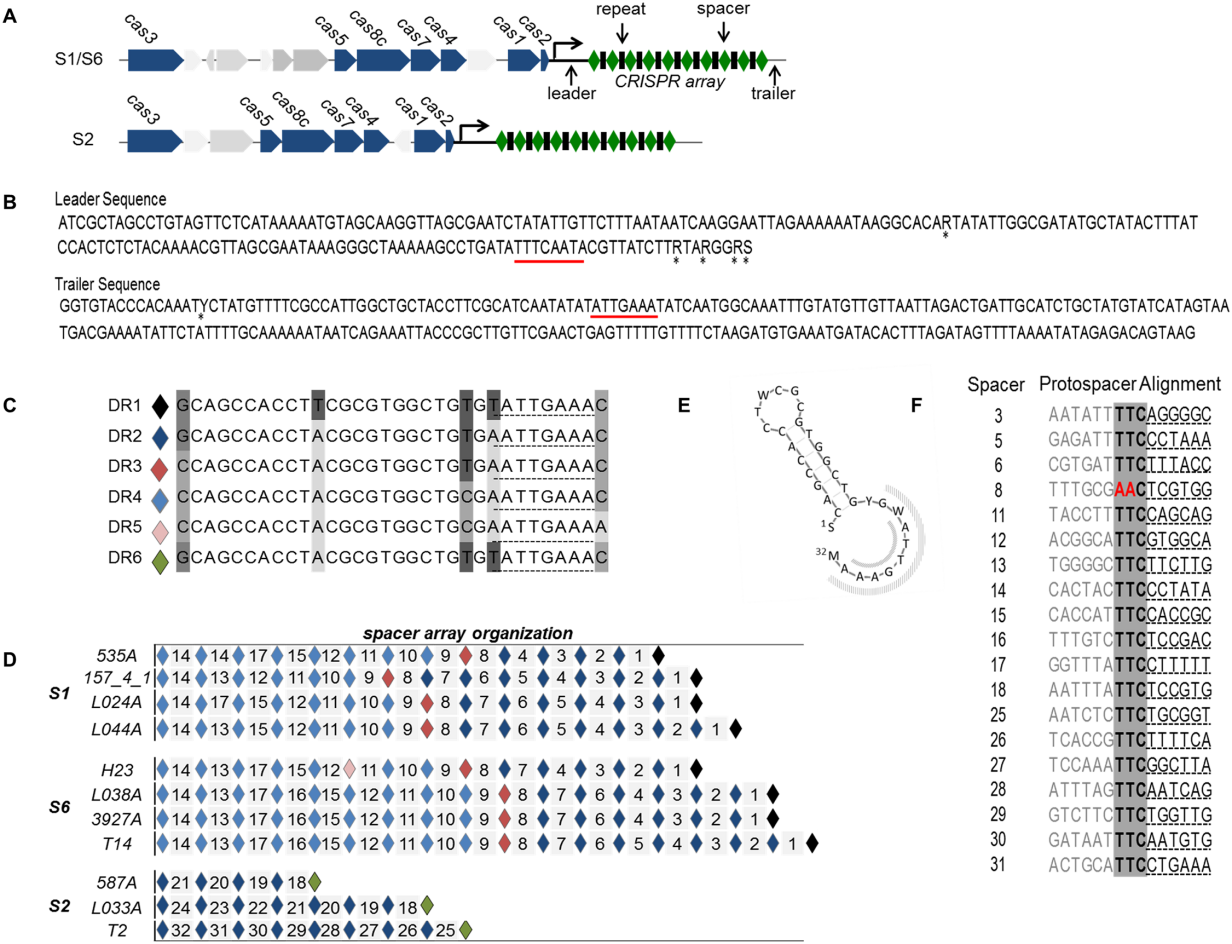
Schematic of CRISPR-Cas systems identified in whole genome sequence analysis of 11 *Mannheimia haemolytica genomes*. Panel A: Cas loci present in serotype 1, 6 and 2 genomes. Panel B: consensus sequence for leader and trailer regions of CRISPR arrays. Underlined regions are complementary to psi-tag. Panel C: Sequences of the 6 unique direct repeats detected in CRISPR arrays. Panel D: CRISPR array spacer and direct repeat organization. Diamonds represent direct repeat sequences while numbered tiles represent spacer sequences. Panel E: 3D structure of direct repeats found in CRISPR arrays. Psi-tag indicated by grey shaded regions. Panel F: sequence alignment of protospacers with suspected protospacer adjacent motif (PAM) highlighted in grey (TTC).

All of the genomes sequenced here contain a single type I-C/Dvulg CRISPR-Cas system (Fig 7A), similar in structure to the those first described by Lawrence et al [5] in *M*. *haemolytica* S2 strains. These are highly conserved within serotype (>98% nucleotide identity) with the most sequence variation occurring between S2 and S1/S6 genomes, although overall pairwise identity across all strains is >95% (S2 Table). The leader and trailer sequences flanking the CRISPR arrays are highly conserved with >98% pairwise identify among strains (Fig 7B), a result not unexpected as these regions are usually highly conserved within a species [40]. There are six unique DR sequences of 32 nucleotides represented among the arrays; these varying at positions 1, 11, 22, 24 and/or 32(Fig 7C). The DR sequences are organized into two blocks in S1 and S6 genomes, each consisting of a unique DR sequence followed by a string of 4–8 identical DR sequences (Fig 7D). In S2 strains only one block of DRs occur, with them being identical to those seen in the first block of S1/S6 arrays. It is unclear why this pattern is present in S1 and S6 strains but not S2. It is possible that similar blocks occur in S2 strains that have larger arrays. Those identified here contain half of the number of spacers observed in S1 and S6 CRISPR arrays.

Direct repeat sequences are target sites for Cas proteins [41] and sequence variation in these regions could affect the regulation of crRNA maturation among these strains. Regulatory elements such as scaRNA and tracrRNAs are described in the Type II CRIPSR system [39], but little has been documented regarding cis or trans elements that may regulate the activates of Type I CRISPR systems. A conserved terminal eight nucleotide motif ((T/A)ATTGAAA) or psi-tag, implicated in Cas protein binding [42] and self-discrimination [43], was identified in the DR sequences and in the leader (in reverse orientation) and the trailer of all CRISPR arrays (Fig 7E). Sequence variability occurs in the region of the DRs, where this motif aligns. It is possible that this psi-tag plays a role in regulation of CRISPR activity in association with altered binding affinity at these locations. The DRs in the arrays examined are homologous (90–100% nucleotide identify) to those occurring in arrays in other Pasteurellaceae members including *Bibersteinia trehalosi* USDA-ARS-USMARC-190, *Mannheimia varigena* USDA-ARS-USMARC-1312 and *Aggregatibacter actinomycetemcomitans* D7S-1 and possessed a secondary structure consistent with those from the CRISPR-Cas I-C/Dvulg subtype [44].

Thirty-two unique spacer sequences (35–37 nt) are present in the CRISPR arrays, with S2 genomes containing fewer spacers (4–8) than S1 (13–15) and S6 (14–17) (Fig 7D). A duplicate spacer was identified in the array of *M*. *haemolytica* 535A and although not common, spacer duplication has been previously identified [45]. The majority of the spacers in the arrays are associated with prophages within the host chromosomes (Table 3). It is possible that these CRISPR spacers are regulating lysogeny, a function of CRIPSR-Cas previously observed in *E*. *coli* [46]. Bacteriophages have developed multiple strategies to avoid CRISPR regulation including mutations within either the protospacer or PAM or through phage encoded anti-CRISPR systems [47,48]. As a result, it is not surprising to see multiple prophages integrated into the S1 and S6 genomes even though these strains contain large CRISPR arrays. Homology searches of the genes present in prophage regions failed to identify previously described phage encoded anti- CRISPR systems [47,48]. Consistent with other type I-C CRSIPR-Cas [49], examination of the upstream sequences of the protospacers revealed a 3nt PAM of GAA conserved in all but one target protospacer sequence (Fig 7F).

**Table 3:** Spacer sequences identified in CRISPR arrays from 11 *Mannheimia haemolytica* genomes.

Spacer	No. of genomes with CRISPR spacer	No. of genomes with sequence outside of spacer	Spacer target^a^	Gene target
1	8	0	n/a	-
2	6	0	n/a	-
3	6	6	prophage	hypothetical
4	8	0	n/a	-
5	8	4	prophage	hypothetical
6	8	8	prophage	putative tail
7	8	0	n/a	-
8	7	2	prophage	regulatory protein Rha
9	6	0	n/a	-
10	3	0	n/a	-
11	8	8	prophage	hypothetical
12	8	5	prophage	hypothetical
13	7	8	bacterial	UDP-N-acetylglucosamine 2-epimerase
14	8	5	bacterial	glycosyl transferases group 1
15	6	7	prophage	tail sheath protein FI
16	3	4	prophage	Mu protein F
17	7	7	prophage	hypothetical
18	2	8	prophage	hypothetical
19	2	0	n/a	-
20	2	0	n/a	-
21	2	0	n/a	-
22	1	0	n/a	-
23	1	0	n/a	-
24	1	0	n/a	-
25	1	7	prophage	DNA helicase
26	1	6	prophage	hypothetical
27	1	8	prophage	head morphogenesis protein
28	1	10	prophage	hypothetical
29	1	10	prophage	hypothetical
30	1	1	prophage	pyruvate kinase
31	1	15	prophage	hypothetical
32	1	0	n/a	-

^a^n/a, target for spacer sequence not identified

Two spacers present in S1 and/or S6 strains, but absent in S2 CRISPR arrays, are complementary to motifs found in genes present within the host chromosome. Approximately 18% of organisms containing CRISPR arrays display self-targeting spacers [41]. However, these are poorly conserved often only being found in a single strain and in association with CRISPR systems that have partial or no activity [41]. An exception to this has been documented in *Francisella novicida*, where a self-targeting CRISPR-Cas system has been found to facilitate immune evasion during infection [50]. In this species the CRISPR system suppresses expression of an immune stimulatory lipoprotein (BLP) that would otherwise trigger the activation of Toll-like Receptor 2-dependant proinflammatory response in the host. Absence of this regulation completely attenuates the bacteria during infection making CRISPR-Cas critical for *F*. *novicida* pathogenesis [50].

The self-targeting spacers here are found in more than one strain and are in association with complete CRISPR systems. Further they are only present in serotypes commonly associated with disease. One spacer targets a glycosyl transferase (F388_04799), the other a UDP-N-acetylglucosamine 2-epimerase (F388_04774). It is likely that these genes code for proteins associated with display of sugar moieties on the cell surface as glycosyl transferases transfer activated sugars to a variety of substrates and UDP-N-acetylglucosamine 2-epimerase is a rate-limiting enzyme in the sialic acid biosynthetic pathway. Sialic acids are prevalent on the surface of vertebrate cells and serve a variety of biological, biophysical and immunological functions [51]. As a result many pathogens, including *Neisseria meningitidis*, *Campylobacter jejuni*, Group B *Streptococcus*, *Pseudomonas aeruginosa* and *Haemophilus influenzae* [52] incorporate sialic acid on their cell surface as a form of molecular mimicry to avoid host immune detection or to aid in adhesion [51,53]. Although it is possible that *M*. *haemolytica*, like *F*. *novicida* is employing CRISPR-Cas to regulate gene expression to avoid host immune responses or to enhance colonization during infection.

### Integrative Conjugative Elements

**Fig 8:**
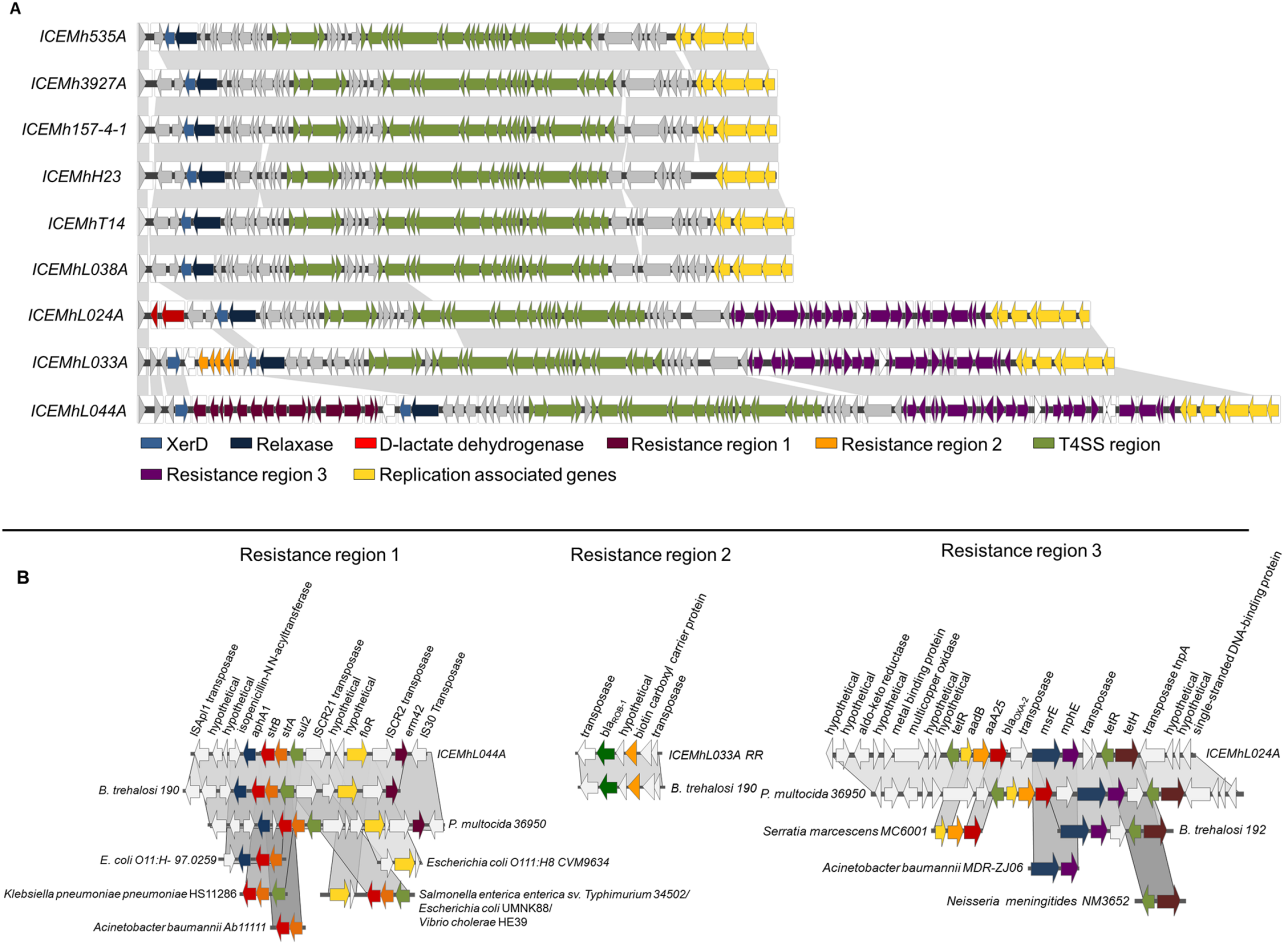
Schematic of putative integrative conjugative elements identified in whole genome sequences of *Mannheimia haemolytica*. Genes are represented as arrows. Grey background indicated regions >94.5% sequence identity. Panel A: proposed ICE genes arrangement. Panel B: resistance gene regions with alignments against cassettes found in other bacterial species.

Putative ICEs were identified in 9 of the 11 genomes sequenced, ranging in size from 46.6 to 81.1 kb (Fig 8A). These elements encode all of the necessary machinery for replication and conjugative transfer, three of which, like ICE*Mh*1 previously identified in *M*. *haemolytica* [10], are active and mobile [54]. Although ICEs integrate into the host genome, under certain conditions they can excise, circularize and replicate. Transfer of ICEs in Gram-negative bacteria typically occurs via conjugation machinery coded for by a type 4 secretion system (T4SS) consisting of a membrane spanning secretion channel and extracellular pilus [26]. After an element has excised from the chromosome, a relaxosome formed by several proteins assembles at the origin of transfer. A relaxase nicks the double stranded circular element and then while maintaining a complex with the single stranded DNA, it is recognized by a coupling protein and is recruited to the T4SS translocation channel. After the DNA is translocated, the relaxase detaches and the element is circularized. Complementary DNA is synthesized and the double stranded element is integrated into the chromosome by an integrase [55]. A copy of the original ICE remaining in the donor undergoes complementary strand synthesis and reintegrates back into the host genome [26]. All of the ICEs identified in the present study are integrated into similar location, in a copy of the tRNA^Leu^ gene and contained a type IV secretion system (T4SS), a coupling protein, an integrase and a relaxase (Fig 8A; S2 Table).

All T4SSs identified belonged to the MPF_G_ family, originally described in ICEHin1056 from *Haemophilus influenzae* [56], but based on public databases prevalent in *Pasteurellaceae* spp. (*Gallibacterium anatis*, *Pasteurella multocida* and *Histophilus somni*) and various Gammaproteobacteria (*Vibrio cholera*, *E*. *coli*, *Pseudomonas aeruginosa*, *Klebsiella pneumonia* and *Salmonella enterica)*. Three distinct sequence profiles for the T4SS region occur with overall nucleotide similarity of 85.8%. However, 6 of the 9 ICEs have a nucleotide similarity of 95.5% across the entire array. This, coupled with their prevalence across genetically diverse isolates suggests that ICEs may have an ancient lineage within this species. None of these ‘common’ ICE profiles contained accessory gene cassettes but contig alignment against previously identified ICE*Mh*1 suggested that they may contain a HipA/B toxin-antitoxin system. Closure of these genomes will be required to conclusively determine if this system is contained within these elements, but the presence of toxin-antitoxin systems could explain why ICEs are being maintained at high abundance within the species even though these elements lack selective antimicrobial resistance (AMR) gene determinants.

Entry exclusion is a trait common to conjugative plasmids [57] that prevents conjugative transfer of MGE into bacterial cells that already contain an element with a closely related transfer apparatus [58]. Two mechanisms for entry exclusion in F plasmids have been presented, one that alters the cell’s outer surface making it less receptive to pilus attachment, with the other mediated by proteins that reside in the inner membrane and prevent DNA entry [58,59]. An exclusion system has been well characterized in the SXT/R391 family of ICEs, mediated by small inner membrane proteins expressed by both the donor (TraG) and recipient cells (Eex) [59]. A TraG homolog (F388_01689) was annotated in the T4SSs here with >80% nucleotide similarity to a similar gene found in ICEs from other *Pasteurellaceae* species (*Mannheimia varigena* USDA-ARS-USMARC-1388:CP006953, *Bibersteinia trehalosi* USDA-ARS-USMARC-190:CP006956, *Pasteurella multocida* 36590:CP003022, *Actinobacillus pleuropneumoniae*; NZ_ADX001000031, *Actinobacillus suis* ATCC 33415:Ga0057047_gi672591300.1, *Haemophilus parasuis* D74:Ga0040714_123, *Haemophilus somnus* 2336:NC_010519). A potential analog to Eex, which is also present in these bacteria, was also identified (F388_01694) that exhibited characteristics similar to the Eex in SXT/R391. These included being localized to the cytoplasm, located in the opposite orientation but adjacent to the *traG* gene and exhibiting amino acid similarity in the N terminal region, but high diversity in the C-terminal region of this protein. An active entry exclusion system in these ICEs has significant implications for the spread of these MGE and is discussed further below.

Only three of the ICEs, ICE*Mh*L024A, ICE*Mh*L044A and ICE*Mh*L033A, contain cassettes with accessory genes, but those that do coded for AMR. These cassettes have high similarity to those previously identified in *Pasteurella multocida* (PRJNA86887) and *Bibersteinia trehalosi* 190 (PRJNA182313) with cassette sections or individual genes also present in strains of *E*. *coli*, *Klebsiella pnuemoniae*, *Salmonella*, *Actinobacter baumannii*, *Serratia marcescens* and *Neisseria meningitides* (Fig 8B). In addition to multiple AMR genes including *aphA1*, *strB*, *strA*, *sul2*, *floR*, *erm42*, *tetH*, *aadB*, *aaA25*, *msrE*, *and mhpE*, a *bla*_ROB-1_ gene was identified in ICEMhL033A with a single nucleotide substitution from C to T at position 370. This single base change generates a premature stop codon rendering this gene non-functional resulting in this strain being sensitive to ampicillin [54].

Although only one of the three S2 strains examined here contained an ICE, it harbours an extensive AMR profile. As S2 strains are prevalent commensals among healthy cattle, the spread of AMR ICEs among these populations may be more significant to the environmental resistome than AMR ICEs in S1 and S6 strains that may be removed from the population through effective antimicrobial therapies or if the host succumbs to disease. All of the AMR containing ICEs here originate from cattle in the USA. Phylogenetic analysis of a larger population incorporating different *Pasteurellaceae* species from different cattle production systems may shed insight as to the origin of these elements. Source tracking of isolates is a common practice to gain insight into AMR development and spread, but with the mobility of these elements among species it may become just as important in the future to track ICEs between and within production systems rather than focusing on AMR at the species or strain level.

The potential for entry exclusion in ICEs from *M*. *haemolytica* may affect AMR dissemination. If ICEs that lack resistance cassettes are widespread among *M*. *haemolytica* populations, entry exclusion could render these strains resistant to the uptake of other ICEs that may harbour AMR genes. This would limit the spread of ICEs in *M*. *haemolytica* populations primarily to clonal dissemination or to those cases where the entry exclusion system was ineffective. If non-AMR ICEs are as prevalent in the *Pasteurellaceae* family as they appear based on this dataset, entry exclusion by these elements could explain why there is relatively low overall occurrence of AMR observed in members the *Pasteurellaceae* family [1,60].

A D-lactate dehydrogenase gene was also identified in ICEMhL024A that is also present within the genome of various Pasteurellaceae species (*Bibersteinia trehalosi*: USDA-ARS-USMARC-190, USDA-ARS-USMARC-192, USDA-ARS-USMARC-188*; Haemophilus parasuis*: 84–17975, KL0318; *Haemophilus somni* 129PT; *Actinobacillus pleuropneumoniae*: JL03, AP76, SH0165; *Mannheimia haemolytica*: M4258, USDA-ARS-USMARC-185, USDA-ARS-USMARC-185, D153). Lactate is a by-product of bacterial fermentation and the L-form of this carboxylic acid is readily metabolized in the liver and tissues of mammals. High levels of the D form lactate can accumulate in the blood of cattle during acidosis; a condition common in feedlot calves fed high grain based finishing diets [61]. It is possible that D-lactate dehydrogenase is being used by *M*. *haemolytica* to detoxify D lactate and use it as an energy source.

A multicopper oxidase is present in a resistance region of all three ICEs exhibiting AMR (Fig 8B). Multiple roles have been proposed for multicopper oxidases that include iron transport and copper resistance [62]. Copper is a trace element added to cattle feed [63], the vast majority of which, with all other heavy metals, is excreted in faeces and urine [64]. It is possible the multicopper oxidase is playing a role in copper resistance as a result of environmental exposure. It is also conceivable that the gene is functioning in iron sequestration, a function linked with pathogenesis in *M*. *haemolytica* [65].

### Conclusion

Multiple factors contribute to the virulence in *M*. *haemolytica*. Comparative analysis of whole genome sequences of pathogenic and non-pathogenic serotypes has highlighted sequence diversity and functional differences in key virulence factors, in particular iron acquisition and outer membrane proteins. Overall, S2 genomes contained fewer intact prophages than S1 and S6 strains, possibly affecting virulence profiles as some phage were shown to harbour genes associated with virulence. It is also possible that CRISPR-Cas is playing a role in the surface expression of sialic acid residues on the surface of S1 and S6 strains contributing to immune evasion or adhesion during infection. Integrative conjugative elements were found in all but 2 strains and are likely playing a role in enhancing host survival in strains through the generation of multidrug resistant cassettes. It is possible that these elements are regulating their dissemination within populations through toxin-antitoxin and entry exclusion systems. This holds implications for the spread of AMR within *M*. *haemolytica* populations. Multiple targets for vaccine design against *M*. *haemolytica* were identified that hold promise towards developing a less antimicrobial dependant strategy to manage this pathogen in agricultural settings.

## Methods

### Isolates

Eleven isolates of *M*. *haemolytica* representing serotypes 1, 2 or 6, from healthy, pneumonic or cattle that succumbed to BRD were subject to whole genome sequencing (Table 1). These were selected from an archive of 532 isolates collected between 2007 and 2012 [4,18,65]. Candidates were selected based on genotypic diversity, antimicrobial resistance profile, and geographical location (i.e., Alberta, Canada; Nebraska, Texas, USA; and Nantes, France. Isolates collected from lung tissue, nasopharyngeal swabs samples or tracheal aspirations were all represented (Table 1).

### DNA Extraction and Sequencing

Phenol: chloroform extraction was used to isolate genomic DNA from overnight cultures grown on Tryptic Soy Agar with blood at 37°C. Briefly, *M*. *haemolytica* culture was suspended in 700 μL of T_10_E_25_ (10mM Tris-HCl pH7.5; 25mM EDTA) and 175 μL of 5M NaCl, 35 μL of 10 mg/mL Proteinase K and 44 μL of 20% SDS were added and the mixture was incubated at 65°C for2 h until cells lysed. The lysed mixture was extracted once with phenol, once with phenol:chloroform:isoamylalcohol (25:24:1) and twice with chloroform. Ammonium acetate (10 M) was added to the final aqueous fraction to achieve a final concentration of 0.5 M, followed by 1 volume of isopropanol to precipitate the DNA. The DNA was gently spooled out, added to a new tube containing ice-chilled 70% ethanol and centrifuged at 10,000 x g for 10 min to obtain a DNA pellet. The supernatant was decanted and the tube was left open to allow the pellet to air-dry. The pellet was suspended in 100 μL of nuclease free deionized water.

Genomic library construction and sequencing for *M*. *haemolytica* H23 was performed by Cofactor Genomics (St Louis, MO). A high-quality draft genome was generated using combined assemblies of Roche 454 single-reads and paired-end reads generated using Illumina Genome Analyzer IIx, with sequencing quality and assembly statistics previously described [66]. Library construction and paired-end sequencing of the remaining10 *M*. *haemolytica* isolates was performed using Roche-454 GS FLX Titanium platform by Genome Québec (McGill University, QC) with draft genomes assembled using Newbler software. Details of the sequencing quality and assembly of these 10 isolates is presented in supplemental material (S3 Table). All 11 genomes were annotated through the NCBI Prokaryotic Genome Annotation Pipeline [67].

### Comparative analysis

Percent sequence identity between strains was determined using sequence alignment of the 11 genomes using Mugsy [68] The UPGMA phylogentic analysis was performed in Geneious v 6.1.8 (created by Bomatters, available from http://www.geneious.com/) using whole genome alignments produced in Mauve [69]. The core, accessory and pan-genomes were calculated using the pan-genomes analysis pipeline (PGAP)[70] with cluster analysis cut-off values set at 85% sequence identity over 90% of the sequence length. The PanGP program [71] was used to analyze the pan-genome profile of the 11 *M*. *haemolytica* genomes with the inclusion of an additional 10 *M*. *haemolytica* strains from public databases (S1 Table). For prophage analysis, contigs were ordered based on alignment against *M*. *haemolytica* strain M42548 using progressive Mauve [69], with concatenated contigs analyzed for prophage using PHAST [72]. Full length prophage sequences were aligned and the Neighbour-Joining tree constructed using MAFFT [73]. The resulting tree was rendered using PHYLIP v. 3.68 as part of the Phylemon2 suite of tools [74]. Genes not annotated by PHAST were examined using PSI BLAST [75] and the integrated microbial genomes system (IMG) [76]. Proposed arrangements for ICEs were constructed by pairwise alignments against ICE*Pmu*1 from *P*. *multocida* [77] and ICE*Mh*1 from *M*. *haemolytica* [10] (GenBank accession numbers: CP003022, CP005383, respectively). Where possible, in-house scripts were used to BLAST unassembled reads against previously assembled ICEs to verify gaps. CRISPR/Cas regions were compiled using the CRISPRdb [78] and alignments of individual prophage, CRISPR-Cas, ICEs and virulence genes were performed using Geneious v 6.1.8. When indicated, reference locus ID originate from sequences in either *M*. *haemolytica* serotype 6 H23 (AOGP00000000), *M*. *haemolytica* M42548 (CP005383) or *M*. *haemolytica M42548* (NC_021082).

## Supporting Information

S1 Fig Percentage of the pan-genome, dispensable genome, core genome or unique genes assigned to categories in the Cluster of Orthologous Groups. (TIF)

S1 Table *Mannheimia haemolytica* strains from public databases used in pan-genome analysis. (DOCX)

S2 Table Percent identities of Cas genes, virulence factors, and integrative conjugative elements (ICEs) associated genes from 11 *Mannheimia haemolytica* isolates. (DOCX)

S3 Table Sequencing and assembly quality for 10 *Mannheimia haemolytica* genomes. (DOCX)
