# Supplementary figures and images for: Comparative Genomic Analysis of Mannheimia haemolytica from Bovine Sources
Source: PLoS One. 2016 Feb 29;11(2):e0149520. doi: 10.1371/journal.pone.0149520 (PMC4771134; doi:10.1371/journal.pone.0149520)

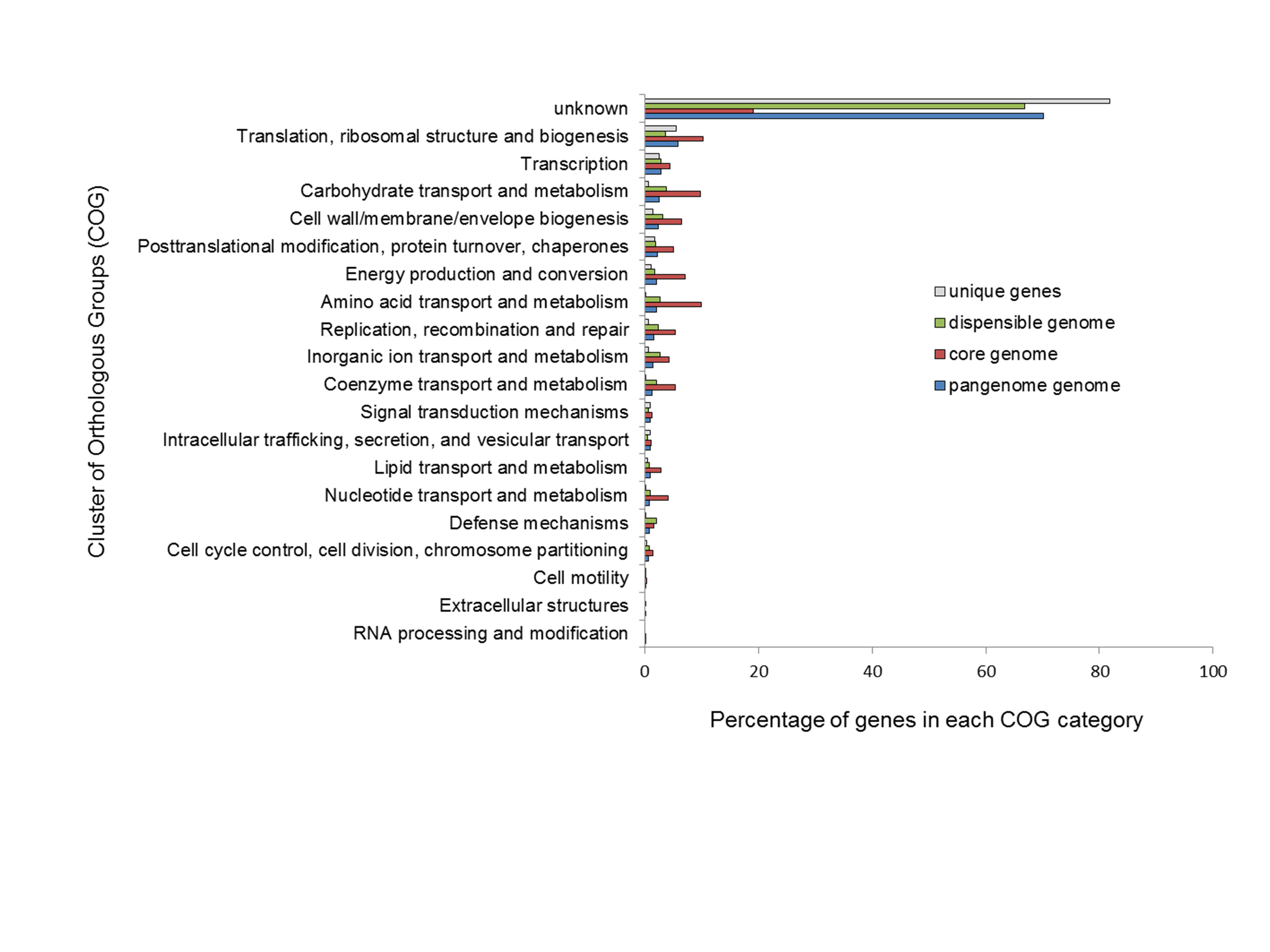

Supplement: S1 Fig — (TIF) [file pone.0149520.s001.tif]
