# Supplementary material for: Comparative Genomic Analysis of Mannheimia haemolytica from Bovine Sources
Source: PLoS One. 2016 Feb 29;11(2):e0149520. doi: 10.1371/journal.pone.0149520 (PMC4771134; doi:10.1371/journal.pone.0149520)
Supplement: S1 Table — (DOCX) [file pone.0149520.s002.docx]

| S1 Table. *Mannheimia haemolytica* strains from public databases used in pan-genome analysis | | | |
| --- | --- | --- | --- |
| Isolate |  | Accession No. | Serotype |
| *M. haemolytica* | D153 | NC_021743.1 | 1 |
| *M. haemolytica* | MhBrain2012 | ATSZ00000000.1 | 1 |
| *M. haemolytica* | D193 | ATSY00000000.1 | 1 |
| *M. haemolytica* | USDA-ARS-USMARC-183 | NC_020833 | 1 |
| *M. haemolytica* | D171 | NC_021738.1 | 2 |
| *M. haemolytica* | D35 | AUNK00000000.1 | 2 |
| *M. haemolytica* | Bovine A2 | NZ_ACZY00000000.1 | 2 |
| *M. haemolytica* | D174 | NC_021739.1 | 6 |
| *M. haemolytica* | D38 | AUNL00000000.1 | 6 |
| *M. haemolytica* | USDA-ARS-USMARC-185 | NC_020834.1 | 6 |
