# Supplementary material for: Comparative Genomic Analysis of Mannheimia haemolytica from Bovine Sources
Source: PLoS One. 2016 Feb 29;11(2):e0149520. doi: 10.1371/journal.pone.0149520 (PMC4771134; doi:10.1371/journal.pone.0149520)
Supplement: S2 Table — (DOCX) [file pone.0149520.s003.docx]

| S2 Table. Percent identities of Cas genes, virulence factors, and integrative conjugative elements (ICEs) associated genes from 11 *Mannheimia haemolytica* isolates  **Supplemental Figure 1** | | | | | | | | | |
| --- | --- | --- | --- | --- | --- | --- | --- | --- | --- |
|  |  |  |  |  | % pairwise identity (% identical sites) | | | | |
| Target | Reference ID^a^ | AA length | % pairwise identity (% identical sites) | sequence length | all | S1/S6 | S1 | S6 | S2 |
| CRISPR-Cas | | | | | | | | | |
| Cas1 | n/a | 337 | 98.8 (97.0) | 1014 | 97.8 (94.9) | 100 (99.9) | 100 (100) | 100(99.9) | 99.9 (99.9) |
| Cas2 | n/a | 97 | 100 (100) | 294 | 100 (100) | 100 (100) | 100 (100) | 100 (100) | 100 (100) |
| Cas2b | n/a | 90 | 100 (100) | 273 |  |  |  |  |  |
| Cas2 vs Cas2b | n/a | 99 | 77.9 (24.2) | 299 | 85.3 (49.5) |  |  |  |  |
| Cas3 | n/a | 762 | 99.5 (98.2) | 2289 | 99.2 (97.1) | 100 (100) | 100 (100) | 100 (100) | 98.3 (97.4) |
| Cas4 | n/a | 224 | 95.6 (89.7) | 675 | 95.0 (88.6) | 99.9 (99.7) | 99.9 (99.9) | 99.9 (99.9) | 100 (100) |
| Cas5 | n/a | 225 | 99.8 (99.1) | 678 | 99.5 (97.6) | 100 (100) | 100 (100) | 100 (100) | 98.4 (97.6) |
| Cas7 | n/a | 287 | 99.6 (99.0) | 864 | 99.4 (97.9) | 100 (100) | 100 (100) | 100 (100) | 99.2 (98.8) |
| Cas8 | n/a | 594 | 99.7 (98.5) | 1785 | 98.6 (95.7) | 99.8 (99.3) | 100 (100) | 99.6 (99.3) | 98.9 (98.4) |
| LPS Pathway |  |  |  |  |  |  |  |  |  |
| 3-deoxy-D-manno-octulosonic-acid transferase WaaA | MHA_0068 | 427 | 99.9 (99.8) | 1281 | 100 (99.9) | 100 (100) | 100 (100) | 100 (100) | 100 (100) |
| possible glycosyltransferase WcaA-like | MHA_0100 | 267 | 99.3 (99.8) | 801 | 99.9 (99.6) | 100 (100) | 100 (100) | 100 (100) | 99.8 (99.8) |
| possible glycosyltransferase WcaA-like | MHA_0101 | 323 | 99.7 (99.1) | 966 | 99.8 (99.3) | 100 (100) | 100 (100) | 100 (100) | 99.6 (99.4) |
| Pasteurellaceae conserved hypothetical protein | MHA_0102 | 395 | 99.9 (99.5) | 1188 | 100.0 (99.8) | 100 (100) | 100 (100) | 100 (100) | 99.9 (99.8) |
| possible glycosyltransferase | MHA_0103 | 261 | 99.9 (99.6) | 786 | 99.9 (99.6) | 100 (100) | 100 (100) | 100 (100) | 99.7 (99.6) |
| possible O-antigen export protein | MHA_0105 | 401 | 99.5 (98.3) | 1207 | 99.9 (99.6) | 100 (99.9) | 100 (99.9) | 100 (100) | 99.9 (99.8) |
| UDP-N-acetylglucosamine 2-epimerase WecB | MHA_0106 | 394 | 99.7 (99.0) | 1185 | 99.9 (99.5) | 100 (100) | 100 (100) | 100 (100) | 99.7 (99.6) |
| heptosyltransferase II (inner core) WaaF | MHA_0191 | 345 | 99.7 (99.9) | 1038 | 100 (99.9) | 100 (100) | 100 (100) | 100 (100) | 100 (100) |
| phosphomannomutase ManB | MHA_0517 | 550 | 99.7 (99.1) | 1653 | 99.5 (97.9) | 100 (100) | 100 (100) | 100 (100) | 98.8 (98.2) |
| UDP-N-acetylglucosamine 2-epimerase WecB | MHA_0521 | 371 | 99.1 (98.4) | 1116 | 98.2 (96.7)^8^ | 98.2 (96.7)^8^ | 100 (100)^4^ | 98.3 (96.7)^4^ | nd |
| ECA biosynthesis protein WecC | MHA_0522 | 423 | 97.4 (95.5)^8^ | 1207 (1272) | 96.4 (93.8)^8^ | 96.4 (93.8)^8^ | 100 (100)^4^ | 98.3 (96.5)^4^ | nd |
| UDP-N-acetyl muramyl pentapeptide phosphotransferase WecA | MHA_0726 | 354 | 100 (100) | 1065 | 100 (100) | 100 (100) | 100 (100) | 100 (100) | 100 (100) |
| lipopolysaccharide chain length determinant protein WzzB | MHA_0727 | 271 | 99.9 (99.6) | 813 | 100 (99.9) | 100 (99.9) | 99.9 (99.9) | 100 (100) | 100 (100) |
| UDP-N-acetyl-D-mannosaminuronic acid dehydrogenase WecC | MHA_0728 | 414 | 99.9 (99.8) | 1245 | 99.9 (99.7) | 100 (99.8) | 100 (100) | 100 (99.9) | 99.8 (99.8) |
| probable fucosamine acetyl transferase | MHA_0729 | 214 | 99.9 (99.5) | 645 | 99.2 (95.7) | 100 (100) | 100 (100) | 100 (100) | 97.1 (95.7) |
| ECA biosynthesis protein WecE | MHA_0730 | 473 | 100 (100) | 1422 | 99.9 (99.7) | 100 (100) | 100 (100) | 100 (100) | 99.9 (99.8) |
| lipopolysaccharide N-acetylglucosaminyltransferase | MHA_0825 | 398 | 100 (100) | 1197 | 100 (99.9) | 100 (100) | 100 (100) | 100 (100) | 100 (100) |
| ADP-glyceromanno-heptose 6-epimerase HldD | MHA_1050 | 308 | 100 (100) | 927 | 100 (99.9) | 100 (100) | 100 (100) | 100 (100) | 99.9 (99.9) |
| UDP-glucose 4-epimerase GalE | MHA_1349 | 338 | 99.8 (99.4) | 1017 | 99.9 (99.8) | 100 (100) | 100 (100) | 100 (100) | 99.9 (99.8) |
| lipid A acyltransferase | MHA_1392 | 316 | 100 (100) | 951 | 99.9 (99.8) | 100 (100) | 100 (100) | 100 (100) | 99.9 (99.9) |
| glycosyltransferase LpsA | MHA_1558 | 263 | 99.8 (99.6) | 792 | 99.9 (99.7)^9^ | 99.9 (99.7)^6^ | 100 (100)^2^ | 99.9 (99.7)^4^ | 100 (100)^3^ |
| lipid A acyltransferase | MHA_1562 | 313 | 99.9 (99.7) | 942 | 99.9 (99.8) | 100 (100) | 100 (100) | 100 (100) | 100 (100) |
| heptosyltransferase II (inner core) WaaF | MHA_1600 | 343 | 99.8 (99.1) | 1032 | 99.9 (99.5) | 100 (99.9) | 100 (100) | 99.9 (99.9) | 99.8 (99.7) |
| O-antigen ligase WaaL | MHA_1845 | 418 | 100 (100)^8^ | 1257 | 100 (100)^8^ | 100 (100)^8^ | 100 (100)^4^ | 100 (100)^4^ | nd |
| dTDP-glucose 4,6-dehydratase RmlB | MHA_1846 | 335 | 100 (100)^8^ | 1008 | 100 (100)^8^ | 100 (100)^8^ | 100 (100)^4^ | 100 (100)^4^ | nd |
| conserved hypothetical protein | MHA_1847 | 193 | 100 (100)^8^ | 582 | 100 (100)^8^ | 100 (100)^8^ | 100 (100)^4^ | 100 (100)^4^ | nd |
| possible LPS sugar transferase | MHA_1848 | 423 | 100 (100)^8^ | 1272 | 100 (100)^8^ | 100 (100)^8^ | 100 (100)^4^ | 100 (100)^4^ | nd |
| LPS chain length determining protein Wzz | MHA_1853 | 375 | 100 (100)^6^ | 1128 | 100 (100)^6^ | 100 (100)^6^ | 100 (100)^2^ | 100 (100)^4^ | - |
| phosphomannomutase ManB | MHA_2240 | 444 | 99.9 (99.5) | 1335 | 99.9 (99.6) | 100 (99.9) | 100 (99.9) | 100 (100) | 99.8 (99.7) |
| D,D-heptose 1-phosphate adenosyltransferase/7-phosphate kinase HldE | MHA_2564 | 475 | 99.7 (99.4) | 1428 | 99.9 (99.7) | 100 (100) | 100 (100) | 100 (100) | 100 (99.9) |
| heptosyltransferase II (inner core) WaaF | MHA_2704 | 345 | 99.4 (98.3) | 1038 | 99.8 (99.3) | 100 (100) | 100 (100) | 100 (100) | 99.6 (99.3) |
| heptosyltransferase II (inner core) WaaC | MHA_2705 | 319 | 99.9 (99.7) | 960 | 99.9 (99.7) | 100 (99.9) | 99.9 (99.9) | 100 (100) | 99.9 (99.8) |
| 3-deoxy-8-phosphooctulonate synthase | MHA_1912 | 284 | 99.7 (98.9) | 855 | 98.6 (95.8) | 100 (100) | 100 (100) | 100 (100) | 97.2 (95.8) |
| 3-deoxy-D-manno-octulosonate 8-phosphate phosphatase | MHA_2646 | 179 | 99.8 (99.4) | 540 | 99.9 (99.8) | 100 (100) | 100 (100) | 100 (100) | 99.9 (99.8) |
| 3-deoxy-D-manno-octulosonate cytidylyltransferase | MHA_0212 | 251 | 100 (100) | 756 | 100 (100) | 100 (100) | 100 (100) | 100 (100) | 100 (100) |
| 3-deoxy-D-manno-octulosonic-acid transferase | MHA_0068 | 426 | 99.9 (99.8) | 1281 | 100 (99.9) | 100 (100) | 100 (100) | 100 (100) | 100 (100) |
| acyl-[acyl-carrier-protein]--UDP-N-acetylglucosamine O-acyltransferase | MHA_0687 | 264 | 100 (100) | 795 | 100 (99.9) | 100 (100) | 100 (100) | 100 (100) | 99.9 (99.9) |
| ADP-L-glycero-D-manno-heptose-6-epimerase | MHA_1050 | 308 | 100 (100) | 927 | 100 (99.9) | 100 (100) | 100 (100) | 100 (100) | 99.9 (99.9) |
| D,D-heptose 1,7-bisphosphate phosphatase | MHA_0543 | 182 | 99.3 (98.4) | 549 | 99.4 (98.4) | 100 (100) | 100 (100) | 100 (100) | 99.5 (99.3) |
| lipid-A-disaccharide kinase | MHA_1351 | 333 | 99.8 (99.4) | 1002 | 99.7 (99.0) | 100 (100) | 100 (100) | 100 (100) | 99.4 (99.1) |
| lipid-A-disaccharide synthase | MHA_0736 | 392 | 99.8 (99.5) | 1179 | 99.9 (99.8) | 100 (100) | 100 (100) | 100 (100) | 99.9 (99.9) |
| Lipopolysaccharide kinase - 3-deoxy-D-manno-octulosonic acid kinase (Kdo/WaaP) | MHA_0049 | 235 | 99.1 (97.9) | 708 | 98.9 (97.5) | 100 (100) | 100 (100) | 100 (100) | 100 (100) |
| phosphoheptose isomerase 583 | MHA_1457 | 193 | 100 (100)^10^ | 582 | 99.9 (99.7)^10^ | 100 (99.8)^8^ | 99.9 (99.8)^4^ | 100 (100)^4^ | 100 (100)^2^ |
| phosphoheptose isomerase 585 | MHA_2736 | 194 | 99.8 (99.5) | 585 | 99.9 (99.7) | 100 (100) | 100 (100) | 100 (100) | 100 (100) |
| rfaE - D-alpha,beta-D-heptose 7-phosphate 1-kinase | MHA_2564 | 475 | 99.7 (99.4) | 1428 | 99.9 (99.7) | 100 (100) | 100 (100) | 100 (100) | 100 (99.9) |
| UDP-2,3-diacylglucosamine hydrolase | MHA_1959 | 233 | 100 (100) | 702 | 100 (100) | 100 (100) | 100 (100) | 100 (100) | 100 (100) |
| UDP-3-O-[3-hydroxymyristoyl] glucosamine N-acyltransferase | MHA_0689 | 341 | 100 (100) | 1026 | 99.6 (98.6) | 100 (100) | 100 (100) | 100 (100) | 99.1 (98.6) |
| UDP-3-O-[3-hydroxymyristoyl] N-acetylglucosamine deacetylase | MHA_2062 | 306 | 99.9 (99.7) | 921 | 99.9 (99.7) | 100 (99.9) | 100 (100) | 99.9 (99.9) | 100 (100) |
| Virulence factors |  |  |  |  |  |  |  |  |  |
| neuraminidase (sialidase) | MHA_1532 | 791 | 99.8 (98.7) | 2377 | 100 (100) | 100 (100) | 100 (100) | 100 (100) | 100 (100) |
| gcp gene - O-sialoglycoprotein endopeptidase | MHA_1559 | 343 | 100 (100) | 1032 | 100 (100) | 100 (100) | 100 (100) | 100 (100) | 100 (100) |
| filamentous hemagglutinin (FhaB) | MHA_0866 | 3214 | 99.6 (99.2) | 9465-9645 | 99.5 (99.0)^7^ | 100 (100)^5^ | 100 (100)^4^ | - | 100 (100)^2^ |
| hemolysin activation/secretion protein FhaC | MHA_0867 | 598 | 98.8 (96.3) | 1797 | 98.7 (96.3) | 99.8 (99.4) | 100 (99.9) | 99.7 (99.4) | 99.2 (98.8) |
| Ssa1 | MHA_2492 | 942 | 93.3 (81.7) | 2253-2829 | 94.0 (83.1) | 99.9 (99.4) | 100 (100) | 99.7 (99.4) | 96.0 (94.6) |
| PlpE | MHA_1514 | 360 | 84.6 (60.6) | 1068-1071 | 88.7 (71.1)^9^ | 100 (100)^7^ | 100 (100) | 100 (100)^3^ | 100 (100)^2^ |
| autotransporter adhesins | MHA_2701 | 2023 | 100 (99.9) | 5658-6072 | 100 (99.9)^8^ | 100 (99.9) | 100 (100) | 100 (100) | nd |
| autotransporter adhesins | MHA_1367 | 1383 | 100 (99.9) | 4152 | 100 (99.9)^8^ | 100 (99.9) | 100 (100) | 100 (100) | nd |
| autotransporters | MHA_0563 | 1398 | 100 (99.9) | 4197 | 100 (99.9)^10^ | 100 (100)^7^ | 100 (100) | 100 (100)^3^ | 100 (100) |
| autotransporters | MHA_2800 | 1503 | 99.9 (99.4) | 4512 | 99.9 (99.8)^9^ | 100 (100) | 100 (100) | 100 (100) | - |
| HmbR1 - hemoglobin receptor | MHA_1639 | 614 | 99.9 (99.8) | 1767-1845 | 99.9 (99.8) | 100 (99.9) | 100 (100) | 100 (100) | 100 (100) |
| HmbR2 - hemoglobin receptor | MHA_2261 | 719 | 99.9 (99.4) | 2160 | 100 (99.9) | 100 (99.9) | 100 (99.9) | 100 (100) | 100 (100) |
| HxuA - hemophore | MHA_1004 | 940 | 98.1 (94.3) | 2817-2823 | 97.9 (94.0) | 100 (100) | 100 (100) | 100 (100) | 98.2 (97.3) |
| TbpA - transferrin-binding protein | MHA_0196 | 937 | 95.8 (83.8) | 2787-2811 | 95.1 (78.5) | 100 (99.9) | 100 (99.9) | 100 (100) | 86.5 (79.8) |
| TbpB - transferrin-binding protein | MHA_0197 | 605 | 82.9 (52.4) | 1752-1767 | 85.0 (57.1) | 99.7 (99.1) | 99.8 (99.5) | 99.8 (99.5) | 75.8 (63.9) |
| FhuE like - ferric hydroximate siderophore receptor | MHA_2388 | 710 | 99.6 (99.2) | 1704-2133 | 99.7 (99.3)^10^ | 100 (100) | 100 (100) | 100 (100) | 100 (100)^2^ |
| ferric hydroximate siderophore receptor | MHA_1541 | 704 | 99.1 (96.7) | 1917-2076 | 99.8 (99.3) | 99.9 (99.7) | 100 (99.9) | 100 (100) | 99.7 (99.6) |
| Fur - ferric uptake regulator | MHA_2790 | 146 | 99.9 (99.3) | 441 | 100 (99.8) | 100 (100) | 100 (100) | 100 (100) | 99.8 (99.8) |
| GS60 - outer membrane lipoprotein | MHA_2734 | 573 | 100 (100) | 1772 | 99.9 (99.8) | 100 (99.9) | 100 (99.9) | 100 (100) | 100 (100) |
| OmpA | MHA_1054 | 378 | 97.7 (93.9) | 1119-1137 | 98.4 (95.9)^10^ | 100 (100)^7^ | 100 (100) | 100 (100) | 98.0 (97.0) |
| LuxS | MHA_2788 | 169 | 99.2 (97.6) | 510 | 98.1 (94.1) | 100 (100) | 100 (100) | 100 (100) | 96.1 (94.1) |
| LktC | MHA_0253 | 184 | 93.3 (79.3) | 456-555 | 97.8 (92.4) | 99.9 (99.8) | 99.9 (99.8) | 100 (100) | 98.5 (97.8) |
| LktA | MHA_0254 | 954 | 94.9 (86.3) | 2862 | 93.7 (82.9) | 100 (100) | 100 (100) | 100 (100) | 95.3 (93.0) |
| LktB | MHA_0255 | 708 | 99.8 (99.2) | 2127 | 98.5 (95.7) | 100 (100) | 100 (100) | 100 (100) | 98.7 (98.0) |
| LktD | MHA_0256 | 478 | 99.9 (99.8) | 1437 | 100(99.9) | 100 (99.9) | 100 (100) | 100 (99.9) | 100 (100) |
| ICE associated genes |  |  |  |  |  |  |  |  |  |
| TraC | n/a | 945 | 98.4 (96.7) | 2838 | 95.1 (89.7) | 96.0 (89.7) | 93.5 (89.7) | 100 (100) | - |
| TraD | n/a | 734 | 98.6 (97.3) | 2202 | 95.5 (91.0) | 96.1 (91.0) | 94.0 (91.0) | 100 (100) | - |
| TraG | n/a | 490-506 | 82.6 (38.7) | 1473-1519 | 93.4 (84.2) | 95.4 (84.2) | 91.4 (84.1) | 99.9 (99.9) | - |
| TraU | n/a | 313 | 97.4 (93.3) | 942 | 96.0 (89.6) | 100 (100) | 94.8 (89.6) | 100 (100) | - |
| TrbI | n/a | 484 | 94.6 (89.3) | 1455 | 92.9 (85.8) | 93.9 (85.8) | 90.6 (85.8) | 100 (100) | - |
| tyrosine recombinase | n/a | 254 | 99.1 (96.1) | 765 | 98.1 (92.9) | 98.4 (93.5) | 96.7 (93.5) | 100 (100) | - |
| tyrosine recombinase 900bp | n/a | 299 | 100 (100) | 900 | 100 (100) | - | - | - | - |
| Relaxase/ TraI | n/a | 516-661 | 96.0 (89.9) | 1551-1986 | 96.1 (89.5) | 97.4 (89.5) | 95.0 (89.6) | 100 (100) | - |
| multicopper oxidase | n/a | 515 | 100 (100) | 1548 | - | - | - | - | - |
| DNA topoisomerase | n/a | 683 | 96.1 (92.2) | 2052 | 92.4 (84.7) | 93.5 (84.7) | 89.8 (84.7) | 100 (100) | - |
| ParB | n/a | 548-549 | 95.8 (91.6) | 1647-1650 | 94.0 (88.1) | 94.9 (88.1) | 92.0 (88.1) | 100 (100) | - |
| pilL | n/a | 209-210 | 94.3 (88.6) | 630-633 | 94.5 (89.0) | 95.3 (89.0) | 92.6 (89.0) | 100 (100) | - |
| ^a^reference locus ID given from M. haemolytica sv. 1 PHL213; accession NZ_AASA00000000.1. n/a, not provided | | | | | | | | | |
