# Supplementary material for: Comparative Genomic Analysis of Mannheimia haemolytica from Bovine Sources
Source: PLoS One. 2016 Feb 29;11(2):e0149520. doi: 10.1371/journal.pone.0149520 (PMC4771134; doi:10.1371/journal.pone.0149520)
Supplement: S3 Table — (DOCX) [file pone.0149520.s004.docx]

| S3 Table. Sequencing and assembly quality for 10 *Mannheimia haemolytica* genomes | | | | | | | |
| --- | --- | --- | --- | --- | --- | --- | --- |
| Strain | No. of reads | No. of bases | Average final read length (bp) | Percentage of reads assembled | No. contigs | Average contig size (bp) | Largest contig size (bp) |
| *M. haemolytica* 157-4-1 | 101,018 | 51,273,525 | 508 | 97.27 | 116 | 22,388 | 187,221 |
| *M. haemolytica* 3927A | 117,738 | 58,869,807 | 500 | 96.89 | 105 | 24,003 | 187,112 |
| *M. haemolytica* 535A | 70,245 | 36,158,101 | 515 | 97.01 | 158 | 16,320 | 139,074 |
| *M. haemolytica* 587A | 105,020 | 53,676,758 | 511 | 97.15 | 111 | 22,535 | 151,288 |
| *M. haemolytica* L024A | 108,125 | 53,014,420 | 490 | 97.06 | 116 | 22,748 | 187,240 |
| *M. haemolytica* L033A | 93,335 | 48,108,457 | 516 | 97.46 | 128 | 20,103 | 144,535 |
| *M. haemolytica* L038A | 81,716 | 41,660,215 | 510 | 96.71 | 134 | 19,406 | 125,011 |
| *M. haemolytica* L044A | 90,156 | 45,273,950 | 502 | 97.47 | 122 | 21,338 | 187,165 |
| *M. haemolytica* T14 | 111,526 | 56,638,572 | 508 | 97.24 | 101 | 25,332 | 173,555 |
| *M. haemolytica* T2 | 90,364 | 45,994,360 | 509 | 97.05 | 107 | 22,668 | 144,065 |
